# Antimicrobial, Antioxidant, and Antityrosinase Activities of *Morina persica* L. and Its Isolated Compounds

**DOI:** 10.3390/molecules29133017

**Published:** 2024-06-26

**Authors:** Rıdvan Özgen, Esen Sezen Karaoğlan, Handan Gökben Sevindik, Hayrunisa Hancı, Cavit Kazaz

**Affiliations:** 1 Department of Pharmaceutical Botany, Faculty of Pharmacy, Inonu University, 44280 Malatya, Turkey; ridvan.ozgen@inonu.edu.tr; 2 Department of Pharmaceutical Botany, Faculty of Pharmacy, Ataturk University, 25240 Erzurum, Turkey; 3 Department of Pharmacognosy, Faculty of Pharmacy, Ataturk University, 25240 Erzurum, Turkey; handansevindik@atauni.edu.tr; 4 Department of Pharmaceutical Microbiology, Faculty of Pharmacy, Ataturk University, 25240 Erzurum, Turkey; hayrunisa.hanci@atauni.edu.tr; 5 Department of Chemistry, Faculty of Science, Ataturk University, 25240 Erzurum, Turkey; ckazaz@atauni.edu.tr

**Keywords:** *Morina persica* L., antimicrobial, antioxidant, antityrosinase

## Abstract

In this study, the isolation of compounds from the aerial parts of *Morina persica* L. and the antimicrobial, antioxidant and antityrosinase activities of various polarity extracts and isolated compounds were investigated. Column chromatography methods were used for isolation. A microdilution method was used to determine antimicrobial activity; Folin-Ciocalteu method was used to determine total phenolic content; DPPH and ABTS radical scavenging- capacity methods were used to determine antioxidant activity; and a mushroom tyrosinase method was used to determine antityrosinase activity. Kaempferol-3-*O*-β-glucopyranoside (astragalin) and quercetin-3-*O*-rutinoside (rutin) were isolated from *M. persica*. The extracts and compounds showed higher activity against *Staphylococcus aureus* and *Enterococcus faecalis* than other tested bacteria. The highest phenolic content, DPPH, and ABTS radical scavenging activity were detected in an ethyl acetate extract at 50 μg/mL concentration. The methanol extract showed the highest antityrosinase effect at 200 μg/mL concentration.

## 1. Introduction

*Morina* (Caprifoliaceae) is a naturally growing genus with aromatic and medicinal properties [1]. Species belonging to this genus are perennial herbaceous plants that are mostly distributed in the South Asian Mountains and the Eastern Mediterranean [2]. There are 15 species of the *Morina* genus belonging to the Caprifoliaceae family [3]. *Morina* species are recorded to contain compounds such as flavonoids, lignans, phenolics, iridoids, and terpenes [1,4,5]. Previous studies have shown that *Morina* species have activities such as antiarthritic, antiasthmatic, antimicrobial, and antioxidant activities [6]. The flowers and thorns of *Morina persica* are used for pain relievers, and the root of *Morina longifolia* is used for skin allergies in folk medicine [7,8].

Infectious diseases caused by microbes are a serious public health concern. Technological developments have allowed the production of new substances with antimicrobial activity. The increasing resistance of pathogens to antibiotics causes serious health problems [9]. Although the discovery of antibiotics has been life-saving, antibiotic resistance is thought to cause the death of millions of people. It is important to develop new strategies against antibiotic resistance, which causes both health and economic problems [10].

Free radicals cause many diseases in humans, such as diabetes, cancer, Alzheimer’s, and Parkinson’s. They also accelerate the spoilage of food. Antioxidants are substances that reduce or prevent these problems caused by free radicals [11].

The tyrosinase enzyme is responsible for melanization in humans and animals. It also causes browning in foods. For this reason, tyrosinase enzyme inhibitors attract the attention of the food and cosmetics industry [12]. Excessive production of melanin in the skin can create hyperpigmented spots and aesthetic problems. Some skin-whitening products that reduce melanogenesis activity and alleviate hyperpigmentation are commercially available. However, tyrosinase inhibitors with a higher efficiency and fewer side effects are needed [13].

Plants are known to contain many phytochemical compounds, have various biological activities, and are used medicinally. There is limited previous research on *Morina persica*. For this reason, it was aimed to isolate the compounds from the aerial parts of *M. persica* L. and determine the antioxidant, antibacterial, and tyrosinase enzymeinhibitor activities of the various extracts and isolated compounds. In isolation studies, polar fractions (water and ethyl acetate) that could contain high amounts of biologically active phenolic compounds and flavonoids are preferred.

## 2. Results

### 2.1. Isolation Results

As a result of the purification studies, kaempferol-3-*O*-β-glucopyranoside (MP1, 34.4 mg) was isolated from the ethyl acetate fraction and quercetin-3-*O*-rutinoside (MP2, 24.3 mg) was isolated from the water fraction.

#### 2.1.1. Kaempferol-3-*O*-β-glucopyranoside (Astragalin) (MP-1)

**Figure 1:**
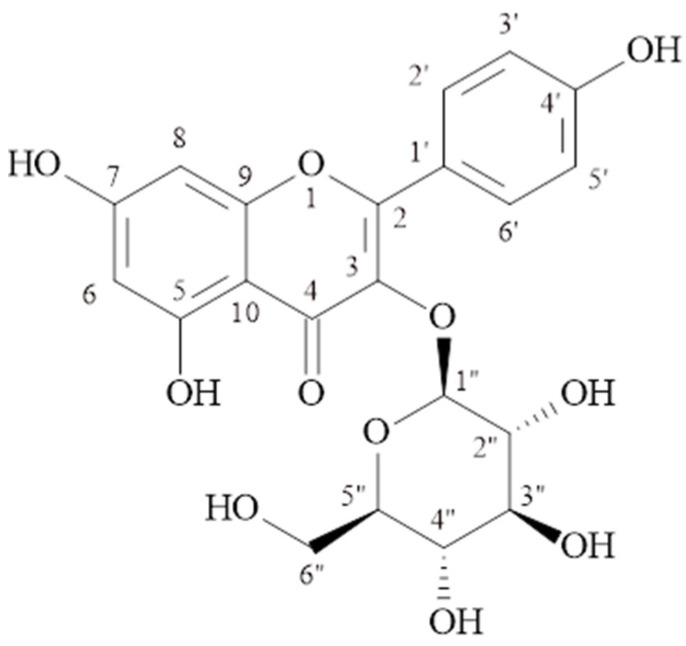
Kaempferol-3-*O*-β-glucopyranoside (Astragalin) (MP-1).

C_21_H_20_O_11_, molecular weight: 449.1078 [M + H]^+^, ^1^H-NMR (δ, ppm); 6.18 (d, *J* = 1.8 Hz. H-6), 6.41 (d, *J* = 1.8 Hz, H-8), 8.01(d, *J* = 8.8 Hz, H-2′, 6′), 6.86 (d, *J* = 9.2 Hz, H-3′, 5′), 5.44 (d, *J* = 7.7 Hz., H-1″), 3.05–3.56 (2″–6″), ^13^C-NMR (δ, ppm); 156.9 (C-2), 133.8 (C-3), 178.1 (C-4), 161.9 (C-5), 99.4 (C-6), 164.8 (C-7), 94.4 (C-8), 157.1 (C-9), 104.7 (C-10), 121.6 (C-1′), 131.6 (C-2′), 115.8 (C-3′), 160.6 (C-4′), 115.8 (C-5′), 131.6 (C-6′), 101.5 (C-1″), 74.9 (C-2″), 77.1 (C-3″), 70.5 (C-4″), 78.2 (C-5″), 61.5 (C-6″). The structural formula is shown in Figure 1.

#### 2.1.2. Quercetin-3-*O*-rutinoside (Rutin) (MP-2)

**Figure 2:**
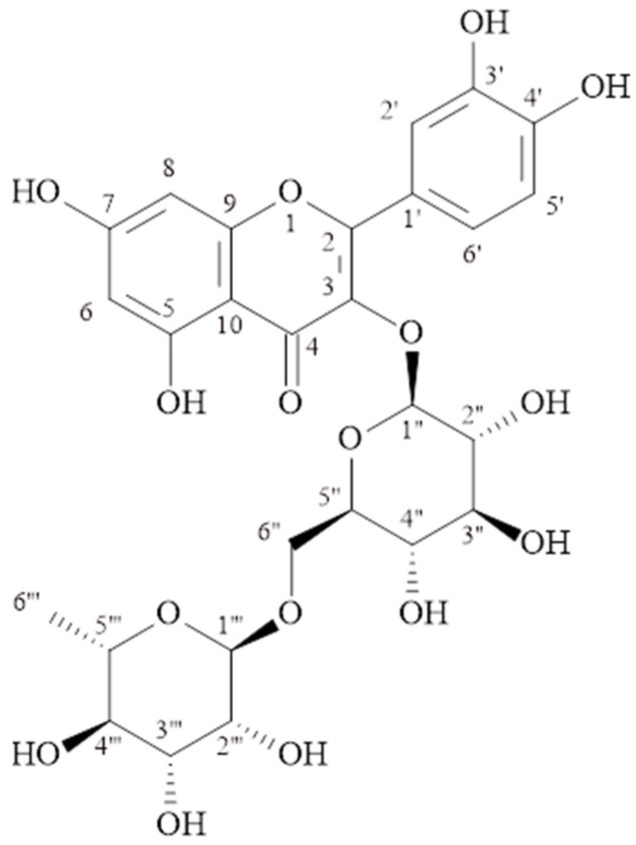
Quercetin-3-*O*-rutinoside (Rutin) (MP-2).

C_27_H_30_O_16_, molecular weight: 611.1602 [M + H]^+^, ^1^H-NMR (δ, ppm); 6.11 (s, H-6), 6.31 (s, H-8), 7.58 (d, *J* = 2.2 Hz, H-2′), 6.79 (d, *J* = 8.7 Hz, H-5′), 7.54 (dd, *J* = 8.4/2.2 Hz, H-6′), 5.0 (d, *J* = 7.7 Hz., H-1″), 3.16–3.55 (H 2″–5″), 3.70 (d, *J* = 10.8, H-6″), 4.42 (s, H-1‴), 3.16–3.55 (H 2‴–5‴), 1.02 (d, *J* = 6.2, H-6‴). ^13^C-NMR (δ, ppm); 158.1 (C-2), 134.3 (C-3), 178.0 (C-4), 161.5 (C-5), 98.6 (C-6), 164.6 (C-7), 93.5 (C-8), 157.3 (C-9), 104.2 (C-10), 121.7 (C-1′), 116.3 (C-2′), 144.4 (C-3′), 148.4 (C-4′), 114.7 (C-5′), 122.2 (C-6′), 103.4 (C-1″), 74.3 (C-2″), 76.8 (C-3″), 70.0 (C-4″), 75.8 (C-5″), 67.2 (C-6″), 101.0 (C-1‴), 70.7 (C-2‴), 70.8 (C-3‴), 72.5 (C-4‴), 68.3 (C-5‴), 16.5 (C-6‴). The structural formula is shown in Figure 2.

### 2.2. Antimicrobial Activity Results

As a result of the study, methanol extract had the strongest effect against *Escherichia coli* (MIC: 4 μg/mL), dichloromethane extract had the strongest effect against *Klebsiella pneumoniae* and *Pseudomonas aeruginosa* (MIC: 16 μg/mL and 512 μg/mL, respectively), and dichloromethane, n-hexane extracts, and MP-1 and MP-2 compounds had the strongest effect against *Staphylococcus aureus* (MIC: 1 μg/mL), and the MP-2 compound had the strongest effect against *Enterococcus faecalis* (MIC: 1 μg/mL). The results are shown in Table 1.

### 2.3. Total Phenolic Compound Quantification Results

**Figure 3:**
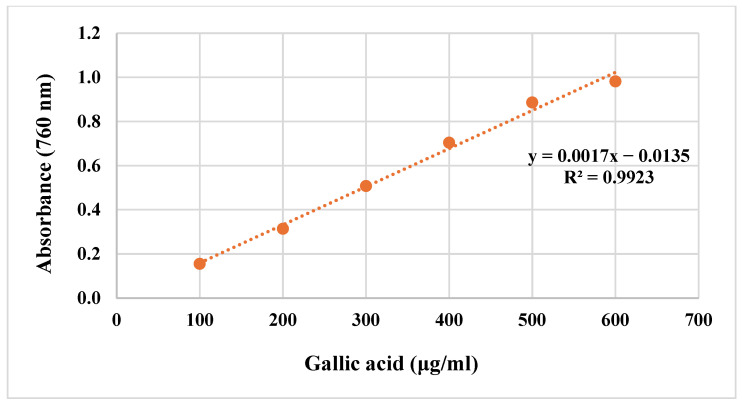
Standard gallic acid graph.

Gallic acid was used as the standard phenolic compound. The total phenolic contents of the extracts were calculated using the equation obtained from the standard gallic acid graph given in Figure 3 and are shown in Table 2. The highest amount of total phenolic compound was found in ethyl acetate extract (169.24 ± 0.229 μg GAE/mg extract).

### 2.4. DPPH Radical Scavenging Study Results

The DPPH radical scavenging activities of extracts, isolated compounds, and trolox at 50 μg/mL concentration are shown in Table 3 in % inhibition. The MP-1 compound showed the highest effect (43.88 ± 0.106 μg/mL), and ethyl acetate extract showed the second (38.80 ± 0.136 μg/mL).

### 2.5. ABTS Radical Scavenger Study Results

The ABTS cation radical scavenging capacities of the extracts, isolated compounds, and trolox at a concentration of 50 μg/mL are shown in Table 4 as % inhibition. Ethyl acetate extract showed the highest effect (98.92 ± 0.341 μg/mL), and the MP-1 compound showed the second highest effect (93.10 ± 0.152 μg/mL).

### 2.6. Tyrosinase Inhibitor Activity Results

The tyrosinase enzyme inhibition percentages of the extracts at 200 μg/mL concentration are shown in Table 5. Additionally, IC_50_ values of MP-1 and MP-2 are given in Table 6. The methanol extract showed the highest percentage of tyrosinase inhibition (%57.14 ± 0.184). The MP-2 compound (115.2 ± 0.8 μg/mL) showed a higher tyrosinase inhibitory effect than the MP-1 (297.9 ± 1.2 μg/mL) compound.

## 3. Discussion

Many plants have medicinal effects and are used in the pharmaceutical industry. Therefore, it is important to investigate the phytochemical and biological activities of new plants. Plant extracts contain many chemical compounds. It is difficult to determine which compound in the plant causes the biological effect. The amount of the effective compound is not the same in every extract. Therefore, dosage adjustment in the extract is complicated. However, dose adjustment is more accurate and reliable in isolated compounds. As far as we have observed, there is not enough research on the *Morina persica* species. Therefore, this research aimed to conduct isolation and various activity studies on *M. persica*.

Two compounds were isolated from the aerial parts of *M. persica* L. The antioxidant, antimicrobial, and tyrosinase inhibitor activities of fractions prepared with varying polarities and isolated compounds were investigated.

In the TLC plate, MP-1 and MP-2 compounds were observed in yellow color in daylight. The observation of a dark stain at UV_254_ nm, dark purple at UV_366_ nm, and a bright yellow color when sprayed with 1% vanillin/H_2_SO_4_ reagent and heated at 110 °C suggested that the compounds may be flavonoids.

The signals at δH 6.18 (d, *J* = 1.8 Hz) and δH 6.41 (d, *J* = 1.8 Hz) ppm observed in the ^1^H NMR spectrum of the MP-1 compound belong to proton numbers 6 and 8 in the molecule, respectively. The signals at δH 6.86 (2H, d, *J* = 9.2 Hz) and δH 8.01 (2H, d, *J* = 8.8 Hz) ppm belong to protons 3′, 5′, and 2′, 6′ of the para-substituted aromatic ring. The signal of the anomeric proton at δH 5.44 ppm (d, *J* = 7.7 Hz) showed the presence of β-configured glucose in the molecule. The signals observed in the ^13^C-NMR spectrum between δC 70.5 and 78.2 ppm also supported the presence of glucose in the molecule. The signal observed at 101.5 ppm is the anomeric carbon signal of glucose. The correlation of the anomeric proton at δH 5.44 (d, *J* = 7.7 Hz, H-1″) ppm with C-3 (δC 133.8 ppm) in the HMBC spectrum showed that glucose was bonded to the aglycone at the third carbon. The results showed that MP-1 is kaempferol-3-*O*-β-glucopyranoside (astragalin) [14,15]. As far as we have observed, kaempferol-3-*O*-β-glucopyranoside was isolated for the first time from the *Morina* species.

The signals at δH 6.11 (s) and δH 6.31 (s) ppm observed in the ^1^H NMR spectrum of the MP-2 compound belong to proton numbers 6 and 8 in the molecule, respectively. Signals at δH 6.79 (d, *J* = 8.7 Hz), δH 7.54 (dd, *J* = 8.4/2.2 Hz), and δH 7.58 (d, *J* = 2.2 Hz) ppm are at 5′, 6′, and 2′ of the quercetin molecule. Anomeric signals at δH 5.0 ppm (d, *J* = 7.7 Hz) and δH 4.42 ppm (s) indicated the presence of two sugars attached to the aglycone. The signals observed in the ^13^C-NMR spectrum between δC 67.2–76.8 ppm also supported the presence of two sugars in the molecule. The signals observed at δC 103.4 ppm (C-1″), δC 101.0 ppm (C-1‴) and δC 16.5 ppm (C-6‴) are the signals of glucose and rhamnose in the molecule. The correlation of the C-3 at δC 134.3 ppm observed in the HMBC spectrum with the H-1″ proton of glucose showed that glucose was bonded to the aglycone at the third carbon. Additionally, in the HMBC spectrum, a correlation was observed between the anomeric carbon (C-1″) of rhamnose and the proton number 6″ of glucose. The compound MP-2 was determined to be quercetin-3-O-rutinoside (rutin) [16,17,18]. Rutin has been isolated from this genus before. As far as we observed, it was isolated from *M. persica* species for the first time in this study.

Microbial diseases pose a threat for public health, and resistance to antibiotics is a serious problem. Therefore, the discovery of new antimicrobials is valuable [9,10]. In our research, methanol extract had the highest antimicrobial effect against *E. coli* (MIC: 4 μg/mL), dichloromethane extract had the highest antimicrobial effect against *K. pneumoniae* and *P. aeruginosa* (MIC: 16 μg/mL and 512 μg/mL, respectively), dichloromethane extract, n-hexane extract, and MP-1 and MP-2 compounds had the highest antimicrobial effect against *S. aureus* (MIC: 1 μg/mL, all of them), and compound MP-2 showed the highest antimicrobial effect against *E. faecalis* (MIC: 1 μg/mL). All extracts and isolated compounds showed significant effects, especially against *S. aureus*. Most of the tested materials were found to be effective against *E. faecalis*. In a previous study, the antibacterial activities of water, acetone, and methanol extracts of *M. persica* against *E. coli*, *P. aeruginosa*, methicillin-resistant *S. aureus*, methicillin-resistant *S. aureus*, and *K. pneumoniae* bacteria were investigated by a microdilution method. Acetone extract and methanol extract showed antibacterial activity against all bacteria [4]. In another study investigating the antibacterial effects of hexane, dichloromethane, and water extracts of *M. persica*, all extracts showed antibacterial effects against *S. aureus*, similar to our study [19]. Taiwo et al. stated that MP-1 is effective against *S. aureus* and *E. faecalis* (MIC values for both: 0.625 µg/mL) [20]. In another study, it was determined that MP-2 was significantly effective against *S. aureus*, *Streptococcus pneumoniae*, *E. coli,* and *Haemophilus influenza* using the disk diffusion method [21].

Antioxidants are substances that prevent pathological conditions caused by oxidative stress and oxidative deterioration in food and pharmaceutical products. In vitro methods are frequently used to determine antioxidant capacity. These methods operate with different kinetic mechanisms [22]. In a previous study, methanol, acetone, and water extracts of the aerial parts of *M. persica* were investigated. Methanol extract showed the highest total phenolic content and the highest ABTS and DPPH radical scavenging capacities [4]. In our study, the ethyl acetate fraction (169.24 ± 0.229 μg GAE/mg extract) had the highest total phenolic content. Additionally, the ethyl acetate fraction (98.92 ± 0.341 μg/mL) and its isolated compound MP-1 (93.10 ± 0.152 μg/mL) showed significant ABTS cation radical scavenging capacities. According to the results of the DPPH radical scavenging method, MP-1 (43.88 ± 0.106 μg/mL) showed the highest effect, followed by ethyl acetate extract (38.80 ± 0.136 μg/mL). However, both were significantly less effective than standard trolox (93.38 ± 0.209 μg/mL). Ethyl acetate extracts are generally rich in flavonoids and phenolic compounds with high antioxidant properties. The highest effect of ethyl acetate extract may be due to this feature.

Tyrosinase is a copper-containing enzyme that initiates melanin synthesis in humans. Excessive accumulation of melanin pigments can cause skin-related problems such as age spots, freckles, browning, and melanoma. In such cases, the tyrosinase enzyme must be inhibited. The discovery of new natural inhibitors is important [23]. In this study, the tyrosinase enzyme inhibitor activities of extracts and isolated compounds were investigated. Methanol extract showed the highest tyrosinase enzyme inhibition activity (57.14%) at a concentration of 200 μg/mL. Additionally, the IC_50_ value of the MP-1 compound was determined as 297.9 ± 1.2 μg/mL, and the IC_50_ value of the MP-2 compound was determined as 115.2 ± 0.8 μg/mL. Methanol is a powerful solvent that dissolves both polar and nonpolar compounds. The reason why the tyrosinase effect of methanol is high may be attributed to the synergistic effect of the compounds it contains. In a previous study, the tyrosinase inhibitory effect of methanol, acetone, and water extracts of *M. persica* was investigated. While the water extract showed tyrosinase inhibitory activity, methanol and acetone extracts were found to be ineffective [4]. In a previous study, the antityrosinase effect of MP-1 isolated from *Euphorbia retusa* was investigated, and the IC_50_ value was found to be 35.46 ± 1.67 μM (standard substance IC_50_: 31.2 ± 1.99 μM) [24]. In another study investigating the tyrosinase enzyme inhibition of MP-2, the IC_50_ values were observed as 55.65 ± 0.98 μg/mL [25]. Some flavonoids have significant tyrosinase inhibitory effects. It may be useful to conduct further studies on this subject [23].

## 4. Materials and Methods

### 4.1. Plant Material

The plant was collected from Konaklı/Erzurum, Turkey in May 2018. A sample of the plant was kept in Atatürk University Biodiversity Application and Research Center Herbarium (AUEF-1175).

### 4.2. Extraction Studies

The plant was collected during the flowering period and dried. The aerial parts of the plant (flowers, leaves, stems, and branches, 342 g) were powdered and macerated with methanol overnight. Then, they were extracted 6 times for 2 h at 40 °C using a reflux cooler and mantle heater. The filtered and combined methanol extract was concentrated and dried in a rotavapor at 40 °C and 120 rpm. The methanol extract (59 g) was dissolved in water and extracted five times in a separating funnel with n-hexane (7.7 g), dichloromethane (10.3 g), and ethyl acetate (2.6 g), respectively, and the obtained extracts were concentrated with the rotavapor. The remainder was the water fraction (34.4 g) [26].

### 4.3. Isolation Studies and Structure Determination

An open column chromatography method was used in the isolation studies. Ethyl acetate extract was applied to the silicagel (Kieselgel 60, 0.063–0.2 mm, Merck, Darmstadt, Germany) column. Dichloromethane:methanol:water (90:10:1, 80:20:2,…, 50:50:5) solvent systems were used. Fractions were evaluated by thin layer chromatography (TLC). Similar fractions were combined. Fractions 19–23 were eluted by the silicagel column (dichloromethane:methanol:water; 90:10:1 and 80:20:2). A precipitate formed in tubes 19–21. The precipitate was washed with methanol and obtained pure MP-1.

The water extract was applied to the silicagel column. Dichloromethane:methanol:water (90:10:1, 80:20:2,…, 30:70:7) solvent systems were used. Fractions were evaluated by TLC. Fractions 231–278 were eluted by the silicagel column (ethyl acetate:methanol:water; 7:2:1, 6:3:1, 5:4:1). The precipitate formed in fractions 8–13 was washed with water. Then, it was applied to the silicagel column (dichloromethane:methanol:water; 90:10:1, 80:20:2,…, 30:70:7). Tubes 72–102 were combined and applied to the silicagel column (dichloromethane:methanol:water; 61:32:7). The compound MP-2 was obtained pure.

The structures of the isolated substances were elucidated by performing ^1^D, ^2^D NMR, and Q-TOF measurements. NMR spectra were taken at the Atatürk University Faculty of Science (Department of Chemistry) and İnönü University Scientific and Technological Research Center (İBTAM). NMR (nuclear magnetic resonance, Bruker, Billerica, MA, USA) spectra were measured on Varian Mercury Plus (400 MHz for ^1^H-NMR and 100 MHz for ^13^C-NMR) spectrometers with TMS as an internal standard. Mass spectra were taken at the Atatürk University Eastern Anatolia Advanced Technology Application and Research Center (DAYTAM). Mass data were recorded on an Agilent 6530 Accurate-Mass Q-TOF LC/MS (Santa Clara, CA, USA).

### 4.4. Antimicrobial Studies

Standard bacterial strains of *Escherichia coli* (ATCC 25922), *Klebsiella pneumoniae* (ATCC 700603), *Pseudomonas aeruginosa* (ATCC 9027), *Staphylococcus aureus* (ATCC 29213), and *Enterococcus faecalis* (ATCC 29212) were used in this study. The antimicrobial activities of the samples were investigated using a microdilution method as recommended by the Clinical and Laboratory Standards Institute [27]. Each sample was double diluted with Mueller Hinton broth (test substances 1024–1 μg/mL and sulfomerazine 3200–3.12 μg/mL). Microorganisms and samples prepared at concentrations of 10^5^ CFU/mL were added to the wells of 96-well sterile plates (50 µL microorganisms and 50 µL samples), and the plates were incubated at 37 °C for 24–48 h. At the end of the incubation, it was visually checked, and the lowest concentrations that did not cause turbidity were determined as the minimum inhibitor concentration (MIC).

### 4.5. Antioxidant Studies

#### 4.5.1. Determination of Total Phenolic Compound Quantity

Gallic acid was used as the standard phenolic compound. First, 1 mL of each gallic acid solution prepared at concentrations of 100, 200, 300, 400, 500, and 600 μg/mL was taken, and each one made up to 23 mL with distilled water. The completed solutions were placed in a conical flask, and 0.5 mL of Folin–Ciocalteu Reagent (FCR) was added and left for 3 min. At the end of this period, 1.5 mL of 2% sodium carbonate (Na_2_CO_3_) solution was added to the mixture. The mixture was stirred in a magnetic stirrer at room temperature for 2 h. Absorbances were recorded at 760 nm against distilled water used as a blank. The absorbance readings were plotted against concentration, and a gallic acid standard graph was obtained [28,29].

To prepare the samples, a stock solution of each sample was prepared at a concentration of 1 mg/mL. Stock solutions were prepared by dissolving the extracts in DMSO. In total, 1 mL of each of these stock solutions was taken and made up to 23 mL with distilled water [30]. The completed solutions were placed in a conical flask, and 0.5 mL FCR was added and left for 3 min. At the end of this period, 1.5 mL of 2% Na_2_CO_3_ solution was added to the mixture. The mixture was stirred in a magnetic stirrer for 2 h at room temperature. Absorbances were recorded at 760 nm against distilled water used as a blank. Measurements were repeated three times. Gallic acid equivalents corresponding to the absorbance of the samples were calculated using the equation obtained from the standard graph. Results were calculated in gallic acid equivalent (GAE) and micrograms (μg).

#### 4.5.2. DPPH Radical Scavenger Capacity Determination

In our study, DPPH radical scavenging capacity determination was made by the Blois method [31]. In total, 1 mM DPPH solution was used as the free radical. Stock solutions of the samples were prepared at different concentrations (10–60 µg/mL). Then, 210 µL of each stock solution and 70 µL of DPPH solution were transferred into each microplate well for measurement. Samples were mixed for 1 min and incubated for 30 min at room temperature in the dark. The absorbance of the samples against ethanol used as a blank was measured at 517 nm. Then, 70 µL DPPH solution and 210 µL ethanol were used as control samples, and trolox was used as a positive control. Percentage inhibition values were calculated using Equation (1) given below.

(1) $$\mathrm{DPPH}\;\mathrm{radical}\;\mathrm{scavenging}\;\mathrm{activity}\;\left(\%\right)=\frac{A\;\mathrm{control}-A\;\mathrm{sample}}{A\;\mathrm{control}}\times 100$$

A_sample_: Absorbance value recorded by adding sample to DPPH solution

A_control_: Absorbance value of control solution

#### 4.5.3. ABTS^+^ Radical Scavenger Capacity Determination

In this study, the ABTS cation radical scavenging capacity of the samples was determined according to the study by Re et al. [32]. To obtain ABTS cation radical, firstly, 2 mM ABTS solution was prepared. ABTS cation radical was obtained by adding 2.45 mM potassium persulfate solution to the prepared solution at a 1:1 ratio. Then, 100 µL of ABTS^•+^ solution and 140 µL of the stock solutions of samples prepared at different concentrations (10–60 µg/mL) were transferred to the wells and shaken for 1 min. In total, 140 µL of ABTS^•+^ solution and 100 µL of phosphate buffer (pH = 7.4, 0.1 M) were used as the control samples. The absorbance of the control sample at 734 nm was 0.700 ± 0.025. After a 30 min waiting period, absorbances were measured at 734 nm against the blank consisting of buffer solution. Trolox was used as the standard compound. The ABTS^•+^ scavenging capacities of the extracts were calculated as a percentage using Equation (2).

(2) $$\mathrm{ABTS}\;\mathrm{radical}\;\mathrm{scavenging}\;\mathrm{activity}\;\left(\%\right)=\frac{A\;\mathrm{control}-A\;\mathrm{sample}}{A\;\mathrm{control}}\times 100$$

A_sample_: Absorbance value recorded by adding sample to ABTS solution

A_control_: Absorbance value of the control sample

### 4.6. Determination of Tyrosinase Enzyme Inhibition

The tyrosinase enzyme inhibitory properties of the samples were tested by modifying Masuda’s method [12]. In this method, where 3,4-dihydroxy-l-phenylalanine (L-DOPA) was used as the substrate, kojic acid was used as a positive control. Then, 40 µL of the sample solution at different concentrations, 40 µL of tyrosinase solution, and 80 µL of phosphate buffer (pH = 6.8) were transferred to 96-well microplate wells and incubated for 10 min at 23 °C. At the end of this period, 40 µL L-DOPA was added to all wells, incubated again at 23 °C for 10 min, and absorbance was measured at 490 nm. The percentage inhibition values were calculated using Equation (3).

(3) $$\%\;\mathrm{Inhibition}\;\mathrm{value}=\frac{\left(A-B\right)-\left(C-D\right)}{\left(A-B\right)}\times 100$$

A: Phosphate buffer and enzyme
B: Phosphate buffer
C: Phosphate buffer, enzyme, and sample
D: Phosphate buffer and sample

## 5. Conclusions

This study aimed to evaluate the various biological activities of the different fractions and isolated compounds of *Morina persica*. It is thought that *M. persica* and its isolated compounds kaempferol-3-*O*-β-glucopyranoside and quercetin-3-*O*-rutinoside can be used as antimicrobial and antioxidant agents, and more detailed studies on this subject may be useful. The data obtained in this research can guide phytochemical and biological activity studies on the *Morina* genus and *M. persica* L. species.

## Figures and Tables

**Table 1:** MIC values of extracts and pure substances (µg/mL).

	*Escherichia coli* (ATCC 25922)	*Klebsiella pneumoniae* (ATCC 700603)	*Pseudomonas aeruginosa* (ATCC 9027)	*Staphylococcus aureus* (ATCC 29213)	*Enterococcus faecalis* (ATCC 29212)
Dichloromethane	64	16	512	1	2
*n*-Hexane	32	64	1024	1	4
Ethyl acetate	32	64	1024	8	64
Aqueous	128	256	1024	4	32
Methanol	4	256	1024	4	8
MP-1	32	1024	1024	1	4
MP-2	128	1024	1024	1	1
Sulfamerazine	1600	1600	1600	6.25	3.12

MIC: Minimum inhibitory concentrations, MP-1: Kaempferol-3-*O*-β-glucopyranoside, MP-2: Quercetin-3-*O*-rutinoside.

**Table 2:** Total phenolic compound amounts of extracts.

Extract	Total Phenolic Compound (μg GAE/mg Extract)
Methanol	49.29 ± 0.105
*n*-Hexane	22.24 ± 0.080
Dichloromethane	53.23 ± 0.152
Ethyl acetate	169.24 ± 0.229
Aqueous	46.71 ± 0.059

Total phenolics (Mean ± SD of three experiments), GAE (Gallic acid equivalents).

**Table 3:** Percentage inhibition values of the DPPH radical scavenging capacity of extracts, isolated compounds, and trolox.

	% Inhibition (50 μg/mL)
Methanol	11.80 ± 0.021
*n*-Hexane	5.50 ± 0.003
Dichloromethane	10.65 ± 0.041
Ethyl acetate	38.80 ± 0.136
Aqueous	10.25 ± 0.066
MP-1	43.88 ± 0.106
MP-2	19.00 ± 0.023
Trolox	93.38 ± 0.209

% Inhibitions (mean ± SD of three experiments), MP-1: Kaempferol-3-*O*-β-glucopyranoside, MP-2: Quercetin-3-*O*-rutinoside.

**Table 4:** Percentage inhibition values of the ABTS cation radical scavenging capacity of extracts, isolated compounds, and trolox.

	% Inhibition (50 μg/mL)
Methanol	40.92 ± 0.198
*n*-Hexane	15.99 ± 0.034
Dichloromethane	28.70 ± 0.148
Ethyl acetate	98.92 ± 0.341
Aqueous	42.88 ± 0.127
MP-1	93.10 ± 0.152
MP-2	68.59 ± 0.211
Trolox	98.71 ± 0.301

% Inhibitions (mean ± SD of three experiments), MP-1: Kaempferol-3-*O*-β-glucopyranoside, MP-2: Quercetin-3-*O*-rutinoside.

**Table 5:** % Tyrosinase enzyme inhibition values of extracts.

	% Inhibition (200 μg/mL)
Methanol	57.14 ± 0.184
*n*-Hexane	31.03 ± 0.197
Dichloromethane	51.72 ± 0.203
Ethyl acetate	20.35 ± 0.136
Aqueous	17.24 ± 0.105
Kojic acid	90.48 ± 0.312

% Inhibitions (mean ± SD of three experiments).

**Table 6:** Tyrosinase enzyme inhibition IC_50_ values of MP-1 and MP-2.

	IC_50_ (μg/mL)
MP-1	297.9 ± 1.2
MP-2	115.2 ± 0.8
Kojic acid	14.6 ± 0.5

IC_50_: Half-maximal inhibitory concentration (mean ± SD of three experiments), MP-1: Kaempferol-3-*O*-β-glucopyranoside, MP-2: Quercetin-3-*O*-rutinoside.

## Data Availability

Data are contained within the article.

## References

[1] Kumar A., Varshney V.K., Rawat M.S.M., Sahrawat S. (2013). Chemical constituents of *Morina* genus: A comprehensive review. Am. J. Essent. Oil. Nat. Prod..

[2] Yuan Q., Zhang J., Yao Z., Zhou Q., Liu P., Liu W., Liu H. (2024). Prediction of potential distributions of *Morina kokonorica* and *Morina chinensis* in China. Ecol. Evol..

[3] Caprifoliaceae Juss., The World Flora Online. http://www.worldfloraonline.org/taxon/wfo-7000000112.

[4] Mocan A., Zengin G., Uysal A., Günes E., Mollica A., Değirmenci N.S., Alpsoy L., Aktumsek A. (2016). Biological and chemical insights of *Morina persica* L.: A source of bioactive compounds with multifunctional properties. J. Funct. Foods.

[5] Zhu Y., Lu Z.P., Xue C.B., Wu W.S. (2009). New triterpenoid saponins and neolignans from *Morina kokonorica*. Helv. Chim. Acta.

[6] Ozgen R. (2023). Pharmacognostic Studies on *Morina persica* L.. Master’s Thesis.

[7] Dolarslan M., Gul E., Dinç B.G., Yalçın D. (2023). Ethnobotanical, Morphological and Ecological Characteristics of *Morina persica* L. (Morinaceae). Science Horizons: Nanotechnology, Plant Diversity, Microbial Insights, and Sustainable Solutions.

[8] Shah A., Abass G., Sharma M.P. (2012). Ethnobotanical study of some medicinal plants from tehsil BudhaL, District Rajouri, (Jammu and Kashmir). Int. Multidiscip. Res. J..

[9] Pettinari C., Pettinari R., Di Nicola C., Tombesi A., Scuri S., Marchetti F. (2021). Antimicrobial MOFs. Coord. Chem. Rev..

[10] Pulingam T., Parumasivam T., Sulaiman A.M., Chee J.Y., Gazzali A.M., Lakshmanan M., Chin C.F., Sudesh K. (2022). Antimicrobial resistance: Prevalence, economic burden, mechanisms of resistance and strategies to overcome. Eur. J. Pharm. Sci..

[11] Halliwell B., Gutterıdge J.M.C. (2015). Free Radicals in Biology and Medicine.

[12] Masuda T., Yamashita D., Takeda Y., Yonemori S. (2005). Screening for tyrosinase inhibitors among extracts of seashore plants and identification of potent inhibitors from *Garcinia subelliptica*. Biosci. Biotechnol. Biochem..

[13] Obaid R.J., Mughal E.U., Naeem N., Sadiq A., Alsantali R.I., Jassas R.S., Moussa Z., Ahmed S.A. (2021). Natural and synthetic flavonoid derivatives as new potential tyrosinase inhibitors: A systematic review. RSC Adv..

[14] Tram N.C.T., Son N.T., Thao D.T., Cuong N.M. (2016). Kaempferol and kaempferol glycosides from *Phyllanthus acidus* leaves. Vietnam J. Chem..

[15] Wei Y., Xie Q., Fisher D., Sutherland I.A. (2011). Separation of patuletin-3-O-glucoside, astragalin, quercetin, kaempferol and isorhamnetin from *Flaveria bidentis* (L.) Kuntze by elution-pump-out high-performance counter-current chromatography. J. Chromatogr. A.

[16] Al-Majmaie S., Nahar L., Sharples G.P., Wadi K., Sarker S.D. (2019). Isolation and antimicrobial activity of rutin and its derivatives from *Ruta chalepensis* (Rutaceae) growing in Iraq. Rec. Nat. Prod..

[17] Petrus J.A., Hemalatha S.S., Suguna G. (2012). Isolation and Characterisation of the Antioxidant Phenolic Metabolites of *Boerhaavia erecta* L. Leaves. J. Pharm. Sci. Res..

[18] Xie Z., Liang Z., Xie C., Zhao M., Yu X., Yang M., Huang J., Xu X. (2014). Separation and Purification of Rosmarinic Acid and Rutin from *Glechoma hederacea* L. using High-Speed Counter-Current Chromatography. Sep. Sci. Technol..

[19] Tasdemir D., Brun R., Perozzo R., Dönmez A.A. (2005). Evaluation of Antiprotozoal and Plasmodial Enoyl-ACP Reductase Inhibition Potential of Turkish Medicinal Plants. Phytother. Res..

[20] Taiwo F.O., Oyedeji O., Osundahunsi M.T. (2019). Antimicrobial and Antioxidant Properties of Kaempferol-3-O-glucoside and 1-(4-Hydroxyphenyl)-3-phenylpropan-1-one Isolated from the Leaves of *Annona muricata* (Linn.). J. Pharm. Res. Int..

[21] Al-Shabibi M.H.S., Al-Touby S.S.J., Hossain M.A. (2022). Isolation, characterization and prediction of biologically active glycoside compounds quercetin-3-rutinoside from the fruits of *Ficus sycomorus*. Carbohydr. Res..

[22] Gulcin I. (2020). Antioxidants and antioxidant methods: An updated overview. Arch. Toxicol..

[23] El-Nashar H.A.S., El-Din M.I.G., Hritcu L., Eldahshan O.A. (2021). Insights on the Inhibitory Power of Flavonoids on Tyrosinase Activity: A Survey from 2016 to 2021. Molecules.

[24] Elgamal A.M., El Raey M.A., Gaara A., Abdelfattah M.A.O., Sobeh M. (2021). Phytochemical profiling and anti-aging activities of *Euphorbia retusa* extract: In silico and in vitro studies. Arab. J. Chem..

[25] Girsang E., Lister I.N.E., Ginting C.N., Sholihah I.A., Raif M.A., Kunardi S., Million H., Widowati W. (2020). Antioxidant and antiaging activity of rutin and caffeic acid. Pharmaciana.

[26] Gözcü S., Uğan R.A., Özbek H., Gündoğdu B., Güvenalp Z. (2024). Evaluation of antidiabetic and antioxidant effects of *Polygonum cognatum* Meisn. and phytochemical analysis of effective extracts. BMC.

[27] (2017). Performance Standards for Antimicrobial Susceptibility Testing, Twenty-Seventh Informational Supplement.

[28] Folin O., Denis W. (1912). On phosphotungstic-phosphomolybdic compounds as colour reagents. J. Biol. Chem..

[29] Slinkard K., Singleton V.L. (1977). Total phenol analysis: Automation and comparison with manual methods. Am. J. Enol. Vitic..

[30] Gulcin I., Tel A.Z., Kirecci E. (2008). Antioxidant, Antimicrobial, Antifungal, and Antiradical Activities of *Cyclotrichium niveum* (BOISS.) Manden and Schen. Int. J. Food Prop..

[31] Blois M. (1958). Antioxidant determinations by the use of a stable free radical. Nature.

[32] Re R., Pellegrini N., Proteggente A., Pannala A., Yang M., Rice-Evans C. (1999). Antioxidant activity applying an improved ABTS radical cation decolorization assay. Free Radic. Biol. Med..

